# Genomic Characterization of Diverse Bat Coronavirus HKU10 in Hipposideros Bats

**DOI:** 10.3390/v13101962

**Published:** 2021-09-29

**Authors:** Ning Wang, Chu-Ming Luo, Xing-Lou Yang, Hai-Zhou Liu, Li-Biao Zhang, Wei Zhang, Bei Li, Yan Zhu, Cheng Peng, Zheng-Li Shi, Ben Hu

**Affiliations:** 1 CAS Key Laboratory of Special Pathogens and Biosafety, Wuhan Institute of Virology, Chinese Academy of Sciences, Wuhan 430071, China; chine.lcm@163.com (C.-M.L.); yangxl@wh.iov.cn (X.-L.Y.); liuhz@wh.iov.cn (H.-Z.L.); zhangwei@wh.iov.cn (W.Z.); libei@wh.iov.cn (B.L.); zhuyan@wh.iov.cn (Y.Z.); pengcheng@wh.iov.cn (C.P.); zlshi@wh.iov.cn (Z.-L.S.); 2 University of Chinese Academy of Sciences, Beijing 100864, China; 3 Shenzhen Institute of Advanced Technology, Chinese Academy of Sciences, Shenzhen 518055, China; 4 Guangdong Key Laboratory of Animal Conservation and Resource Utilization, Guangdong Public Laboratory of Wild Animal Conservation and Utilization, Institute of Zoology, Guangdong Academy of Science, Guangzhou 510260, China; zhanglb@giz.gd.cn

**Keywords:** coronavirus, alphacoronavirus, bat coronavirus HKU10, interspecies transmission

## Abstract

Bats have been identified as natural reservoirs of a variety of coronaviruses. They harbor at least 19 of the 33 defined species of alpha- and betacoronaviruses. Previously, the *bat coronavirus HKU10* was found in two bat species of different suborders, *Rousettus leschenaultia* and *Hipposideros pomona*, in south China. However, its geographic distribution and evolution history are not fully investigated. Here, we screened this viral species by a nested reverse transcriptase PCR in our archived samples collected over 10 years from 25 provinces of China and one province of Laos. From 8004 bat fecal samples, 26 were found to be positive for bat coronavirus HKU10 (BtCoV HKU10). New habitats of BtCoV HKU10 were found in the Yunnan, Guangxi, and Hainan Provinces of China, and Louang Namtha Province in Laos. In addition to *H. pomona*, BtCoV HKU10 variants were found circulating in *Aselliscus stoliczkanus* and *Hipposideros larvatus*. We sequenced full-length genomes of 17 newly discovered BtCoV HKU10 strains and compared them with previously published sequences. Our results revealed a much higher genetic diversity of BtCoV HKU10, particularly in spike genes and accessory genes. Besides the two previously reported lineages, we found six novel lineages in their new habitats, three of which were located in Yunnan province. The genotypes of these viruses are closely related to sampling locations based on polyproteins, and correlated to bat species based on spike genes. Combining phylogenetic analysis, selective pressure, and molecular-clock calculation, we demonstrated that Yunnan bats harbor a gene pool of BtCoV HKU10, with *H. pomona* as a natural reservoir. The cell tropism test using spike-pseudotyped lentivirus system showed that BtCoV HKU10 could enter cells from human and bat, suggesting a potential interspecies spillover. Continuous studies on these bat coronaviruses will expand our understanding of the evolution and genetic diversity of coronaviruses, and provide a prewarning of potential zoonotic diseases from bats.

## 1. Introduction

Coronaviruses (CoVs) are enveloped, non-segmented positive-strand RNA viruses, with a genome ranging from 26–32 kb [1]. According to the nomenclature and taxonomy recently released by the International Committee for Taxonomy of Viruses (ICTV), CoVs are classified into four genera, Alpha-, Beta-, Gamma- and Deltacoronavirus, within the *Orthocoronavirinae* subfamily, *Coronaviridae* family, *Cornidovirineae* suborder, and *Nidovirales* order [2]. All coronaviruses share a similar genome organization and discontinuous transcription mechanism [3]. Two overlapping open reading frames, (ORF)1a/b, occupying 2/3 genome at the 5′ end, encode polyproteins which are cleaved into 15 or 16 nonstructural proteins (nsp1 or nsp2 through nsp16) in host cells. The structural proteins spike (S), envelope (E), membrane (M) and nucleocapsid (N) are expressed from a nested set of 3′ co-terminal subgenomic messenger RNAs (sg mRNAs).

CoVs infect various animals, including but not limited to pig, cat, bird, bat and human. While mammal-infecting CoVs can be found in all four genera, bat CoVs are considered as the genetic source of *Alphacoronavirus* and *Betacoronavirus*, and avian CoVs are considered as the genetic source of *Gammacoronavirus* and *Deltacoronavirus*. [4]. The seven human coronaviruses discovered to date all belong to *Alphacoronavirus* and *Betacoronavirus*, and five of them are phylogenetically related to, or even originate from CoVs circulating in bats [5,6,7,8,9,10,11]. 

CoVs are known to possess high mutation rates and recombination rates, which may allow them to adapt to new hosts and ecological niches [12]. The spike protein recognizes and binds to cellular receptor and then mediate CoV entry. It determines the host specificity of CoVs together with the cellular receptor and is essential for crossing the species barrier. Interspecies transmission of CoVs causes emerging zoonotic diseases that pose a threat to public health. Notably, three of them, including severe acute respiratory syndrome coronavirus (SARS-CoV) [13,14,15], Middle East respiratory syndrome coronavirus (MERS-CoV) [16] and SARS-CoV-2 [11], have caused severe respiratory diseases with high mortality in humans. Previous studies revealed that SARS-CoV originates from bats [5,6,17], while MERS-CoV is phylogenetically associated with the varieties of coronaviruses found in vesper bats [7,10,18], and SARS-CoV-2 likely originates in wildlife, including bats and pangolins. In addition, the swine acute diarrhea syndrome coronavirus, which caused heavy losses on pig farms in recent years, was the result of the spillover of a bat alphacoronavirus [11,19,20,21]. These data indicate that bat coronaviruses probably pose high spillover potentials.

Previous studies identified a bat coronavirus species, *bat coronavirus HKU10* (BtCoV HKU10), from Guangdong Province and Hong Kong of China, and southeast Asian countries, such as Laos and Thailand [22,23,24,25]. These viruses were from two bat species belonging to different suborders, *Hipposideros pomona* and *Rousettus leschenaultia*. Analysis based on positive selection and molecular-clock calculation suggested that BtCoV HKU10 was probably a recent interspecies transmission from *R. leschenaultia* to *H.* *pomona* [25]. In China, Rousettus bats are distributed only in southern regions, while Hipposideros bats are widely found throughout China. In order to better understand the geographic distribution, evolution, and interspecies transmission potential of these viruses, we performed surveillance of this virus in wider geographic areas in our archived samples collected from 25 provinces of China and one province of Laos. Diverse BtCoV HKU10 strains were discovered in south and southwest China and Louang Namtha province of Laos, but not in the northern China. We sequenced the full-length genomes of 17 newly detected HKU10 strains. Phylogenetic analysis, positive selection and molecular-clock calculation demonstrated that interspecies transmission of BtCoV HKU10 may have occurred from *H. pomona* to *R. leschenaultia*, which is opposite to previous report. Our study supports the theory that *H. pomona* is the natural reservoir of BtCoV HKU10, and Yunnan is the geographical source of these viruses.

## 2. Materials and Methods 

### 2.1. Ethics Statement

All sampling procedures were performed by veterinarians with approval from Animal Ethics Committee of the Wuhan Institute of Virology (WIVH05210201, approved on 9 July 2012). The study was conducted in accordance with the Guide for the Care and Use of Wild Mammals in Research of the People’s Republic of China.

### 2.2. Sampling

Bat samplings were conducted from September 2006 to June 2016, as described previously [17,26]. Bat fecal swab and pellet samples were collected at different seasons in 25 provinces in China and one province in northern Laos adjacent to Yunnan province of China.

### 2.3. RNA Extraction, PCR Screening and Sequencing

Viral RNA was extracted from 200 μL of fecal swab or pellet samples with High Pure Viral RNA Kit (Roche Diagnostics GmbH, Mannheim, Germany) as per the manufacturer’s instructions. RNA was eluted in 50 μL of Elution buffer, aliquoted, and stored at −80 °C. A one-step heminested RT-PCR (Invitrogen, San Diego, CA, USA) (Table S1), targeting a 440 nucleotide (nt) fragment of RNA-dependent RNA polymerase (RdRp), was employed to detect the presence of coronavirus sequences, as described previously [27]. PCR products were gel purified and sequenced with an ABI Prism 3730 DNA analyzer (Applied Biosystems, Foster City, CA, USA). Alternatively, the PCR products were cloned into pGEM-T Easy Vector (Promega, Madison, WI, USA) for sequencing. The positive samples in this study were classified using the abbreviated name of sampling location followed by sample ID (e.g., YN3723).

To confirm the bat species of an individual sample, we performed PCR to amplify the cytochrome b (Cytob) or NADH dehydrogenase subunit 1 (ND1) gene using DNA extracted from the feces or swabs [28,29]. The gene sequences were assembled excluding the primer sequences; BLASTN was used to identify host species based on the most closely related sequences with highest query coverage and a minimum identity of 95%. 

### 2.4. Sequencing of Full-Length Genomes

Full genome sequences were determined as previously reported [26]. Briefly, sequences were determined by one-step PCR (Invitrogen, San Diego, CA, USA) amplification with degenerate primers designed on the basis of multiple alignment of available alphaCoV sequences deposited in GenBank, and then amplified with SuperScript IV Reverse Transcriptase (Invitrogen, San Diego, CA, USA) and Expand Long Template PCR System (Roche Diagnostics GmbH, Mannheim, Germany) with specific primers (Figure S1 and Table S1). For the reverse transcription, the reaction mix contained 1 μL of 2 μM gene-specific reverse primer, 1 μL of 10 mM dNTP mix, 5 μL of RNA and 6 μL of nuclease-free water, The mixture annealed at 65 °C for 5 min and then incubated on ice for 2 min. Then, we added 4 μL of 5× SSIV buffer, 1 μL of 100 mM DTT, 1 μL of RNase inhibitor and 1 μL of SuperScript IV reverse transcriptase. Mixed and incubated the mixture at 50 °C for 10 min and inactivated at 80 °C for 10 min. For long template PCT, the 50 μL reaction mix contained 5 μL PCR buffer 2, 500 μM of dNTP, 300 nM of each primer, 0.75 μL of Expand long template enzyme mix, and 5 μL of reverse transcription product. The amplification was performed as follows: 94 °C 5 min followed by 10 cycles consisting of 94 °C 10 s, 50 °C 30 s, 68 °C 10 min, then 25 cycles of 94 °C 10 s, 50 °C 30 s, 68 °C 10 min (+20 s for each cycle), and final elongation at 68 °C for 7 min. PCR products were gel purified and sequenced. Sequences of the 5′ and 3′ genomic ends were obtained by 5′ and 3′ RACE (SMARTer RACE 5′/3′ Kit; Clontech, Mountain View, CA, USA), respectively.

### 2.5. Genome Analysis

Preliminary sequences were assembled using DNAStar lasergene V7 (DNAStar, Madison, WI, USA). Putative open reading frames (ORFs) and deduced amino acid (aa) sequences were predicted using the NCBI’s ORF Finder (https://www.ncbi.nlm.nih.gov/orffinder/ accessed on 29 September 2017) with a minimal ORF length of 150 nt, followed by manual inspection. The sequences of the 5′ untranslated region (5′-UTR) and 3′ untranslated region (3′-UTR) were defined, and the leader sequence, the leader and body TRSs were illustrated as previous described [30]. Phylogenetic trees based on nt or aa sequences were constructed using the Maximum Likelihood algorithm with bootstrap values determined by 1000 replicates in the MEGA 6 software package [31]. Full-length genome sequences detected in this study were aligned with those of previously reported BtCoV HKU10 using MUSCLE [31]. The aligned sequences were scanned for recombination events by using Recombination Detection Program (RDP) [32]. The potential recombination events suggested by strong *p* values (<10–20) were further confirmed using similarity plot and bootscan analyses implemented in Simplot 3.5.1 [33]. The number of synonymous substitutions per synonymous site, Ks, and the number of nonsynonymous substitutions per nonsynonymous site, Ka, for each coding region were calculated using the Ka/Ks calculation tool of Norwegian Bioinformatics Platform (http://services.cbu.uib.no/tools/kaks accessed on 22 October 2017) with default parameters [34].

### 2.6. Virus Isolation

The virus isolation was performed as previously described [5]. Briefly, Vero E6 monolayer was maintained in DMEM medium supplemented with 10% fetal bovine serum (FBS). Fecal supernatant was acquired via gradient centrifuge and then added to Vero E6 cells, and 1:10 diluted in DMEM. Cells were incubated at 37 °C for 1 h and the inoculum was then replaced by fresh DMEM containing 2% FBS and the antibiotic–antimycotic (Gibco, Grand Island, NY, USA). Cells were incubated for another 5 days and checked daily for cytopathic effect. Three passages were carried. Both culture supernatant and cell pallet were examined for CoV by RT-PCR [35].

### 2.7. Estimation of Divergence Time

Divergence time was calculated using RdRp gene sequence and the Bayesian Markov chain Monte Carlo (MCMC) approach as implemented in BEAST (version 2.4.4) as described previously [25]. One parametric model (constant size) and one nonparametric model (Bayesian Skyline with five groups) for tree priors were used for the inference. Analyses were performed with the SRD06 substitution model using both strict and relaxed (uncorrelated lognormal (Ucld) and uncorrelated exponential (Uced)) molecular clock. The MCMC run was 1 × 10^9^ steps long, with sampling every 1000 steps. Convergence was assessed on the basis of the effective sampling size after a 10% run-in using Tracer software version 1.6. The mean time of the most recent common ancestor (tMRCA) and the highest posterior density regions at 95% (HPD) were calculated. Bayes Skyline under a relaxed-clock model with Uced was adopted for making inferences, as Bayes factor analysis showed it fit the data better than other models tested. The trees were summarized in a target tree by the Tree Annotator program included in the BEAST package by choosing the tree with the maximum sum of posterior probabilities (maximum clade credibility) after a 10% burn-in.

### 2.8. Cell Lines

Bat primary or immortalized cells (*Rhinolophus sinicus* kidney immortalized cells, RsKT; *Rhinolophus sinicus* lung primary cells, RsLu4323; *Rhinolophus sinicus* brain immortalized cells, RsBrT; *Rhinolophus affinis* kidney primary cells, RaK4324; *Rousettus leschenaultia* Kidney immortalized cells, RlKT; *Hipposideros pratti* lung immortalized cells, HpLuT) generated in our laboratory were all cultured in DMEM/F12 with 15% FBS [26]. *Pteropus alecto* kidney cells (Paki) was maintained in DMEM/F12 supplemented with 10% FBS [36]. Human cells (Hep-2, A549, Calu-3, H292, Caco-2, Huh-7, Hela, HEK-293T, RD, CCF-STTG1, THP-1), bat lung epithelial cells (Tb1-Lu), and other mammalian cells (LLC-ML2, Vero, BHK21, MDCK, FK, SIEC, NIH-3T3, V79) originated from American Type Culture Collection (ATCC, www.atcc.org accessed on 18 September 2021) were maintained according to the recommendations. 

### 2.9. BtCoV HKU10 Spike-Mediated Pseudovirus Cell Tropism Screening

Retroviruses pseudotyped with the spike from Ro-BtCoV HKU10, YN7560 and MERS-CoV were used to infect human, bat or other mammalian cells in 96-well plates. The pseudovirus particles were confirmed with Western blotting and negative-staining electron microscopy. Pseudovirus containing supernatants were analyzed and transferred to PVDF membrane. HA tag antibodies (Proteintech, Wuhan, China) and P24 antibodies (Keyuananbo, Wuhan, China) were used to detect recombinant S proteins with a C-terminal HA tag and HIV P24 protein, respectively. Pseudovirus containing supernatants were 0.45 μm filtered and centrifuge at 128,000 g for 2 h, and then resuspend the pellet. We loaded 5 μL of purified pseudoviruses into the grids and incubated for 3 min, and 5 μL of 1% phosphotungstic acid was applied for negative staining. Pseudovirus particles were examined in a H-7000FA transmission electron microscope. The production process, measurements of infection and luciferase activity were conducted as described previously [7,37].

### 2.10. Nucleotide Sequence Accession Numbers

The sequences of viral genomes determined in this study have been submitted to GenBank sequence database under accession numbers MN477899 to MN477915.

## 3. Results

### 3.1. Prevalence of BtCoV HKU10

**Figure 1:**
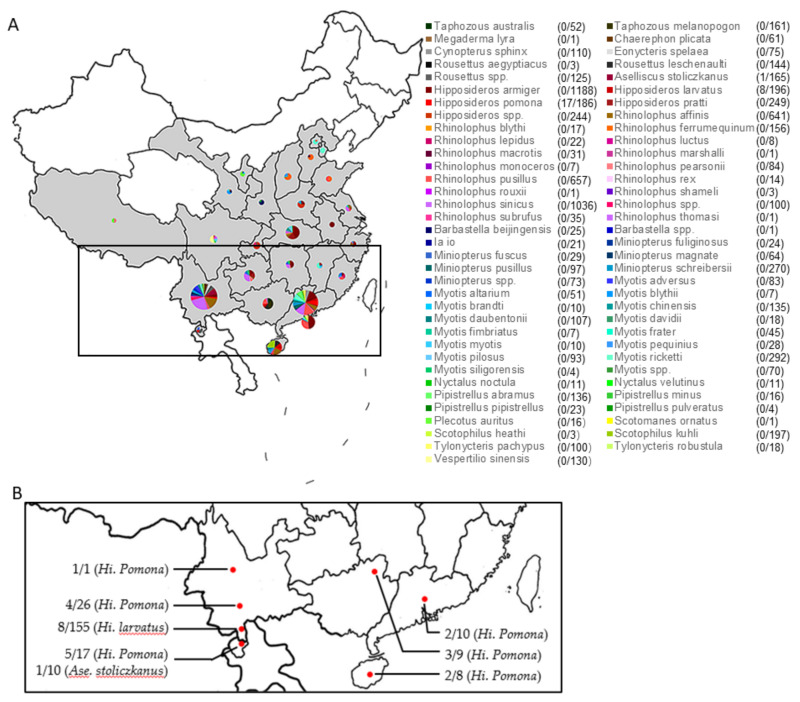
Locations of sampling (**A**) and BtCoV HKU10 positives (**B**). Sampling locations are in gray and bat species are listed in color. BtCoV HKU10 positives are marked in square (**A**) and in red dot (**B**).

A total of 8004 fecal specimens were collected from 25 provinces in China and Louang Namtha province in Laos (Figure 1A). These bats belonged to 69 bat species of 6 bat families according to morphological or molecular identification. By RT-PCR and sequencing, we found 26 were positive for BtCoV HKU10 (Figure 1B and Supplementary Table S2). All positive samples were from three bat species: *Aselliscus stoliczkanus* (1/165), *Hipposideros larvatus* (8/196), and *H. pomona* (17/186) collected in southern and southwestern provinces of China (Yunnan, Guangxi, Guangdong and Hainan) and Louang Namtha province of Laos. We did not find any BtCoV-HKU10 or related viruses from 144 *R. leschenaultia* samples. Neither cytopathic effect nor viral replication was detected, indicating the failure of virus isolation. 

### 3.2. Genomic Characterization of Different BtCoV HKU10 Strains

**Figure 2:**
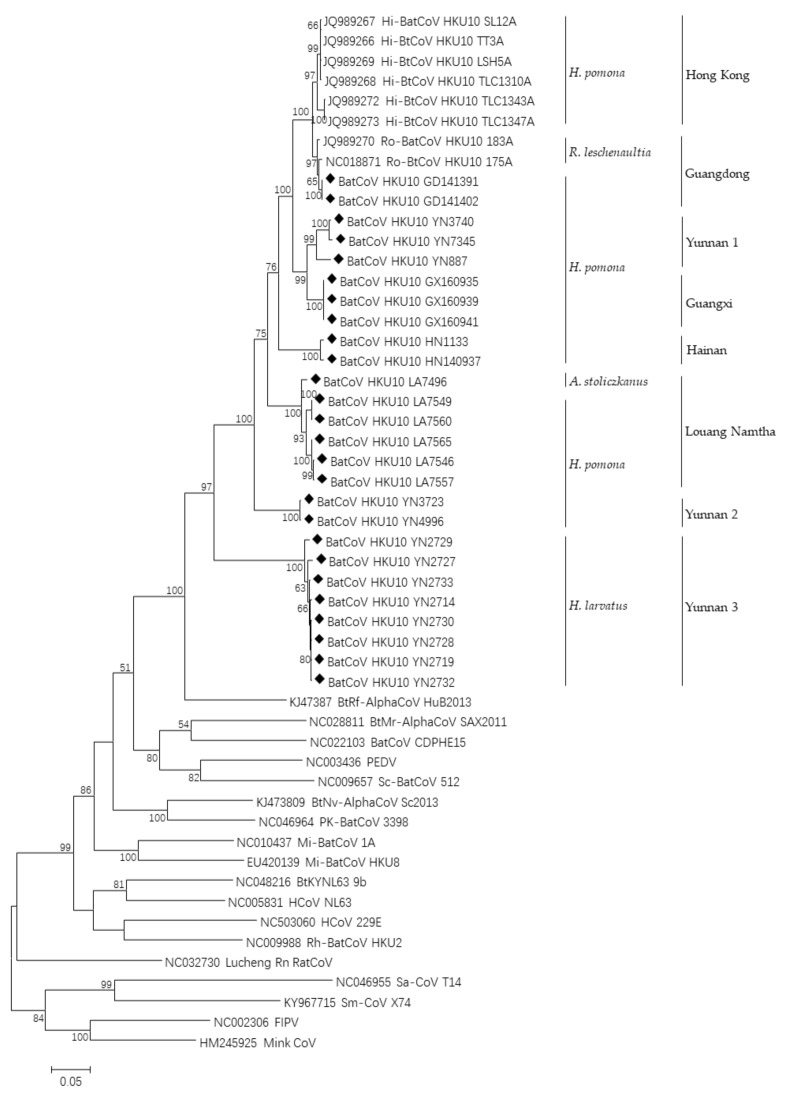
Phylogenetic analysis of BtCoV HKU10 detected in bats. Full-length RdRp genes of viruses detected in this study were aligned with those of published BtCoV HKU10 and representative CoV strains. The tree was constructed by the maximum-likelihood method with bootstrap values determined with 1000 replicates. The scale bar indicates the estimated number of substitutions per 20 nucleotides. Filled diamonds indicate the CoVs detected in this study. FIPV, feline infectious peritonitis virus; PEDV, porcine epidemic diarrhea virus.

We sequenced the complete *RdRp* genes of all 26 strains. These sequences shared high identity to known BtCoV HKU10 (nt: 81.8–99.2%, deduced amino acid: 91.4–99.7%). Phylogenetic analysis based on the *RdRp* sequences indicated that the newly detected viruses, together with the known BtCoV HKU10 strains, formed eight lineages closely associated with the sampling locations (Figure 2). Lineages 1 and 2 were previously reported from Hong Kong and Guangdong, respectively, while the other six were newly discovered. Three of them were from Yunnan province and one each was from Hainan, Guangxi and Laos, respectively (Figure 2). 

**Figure 3:**
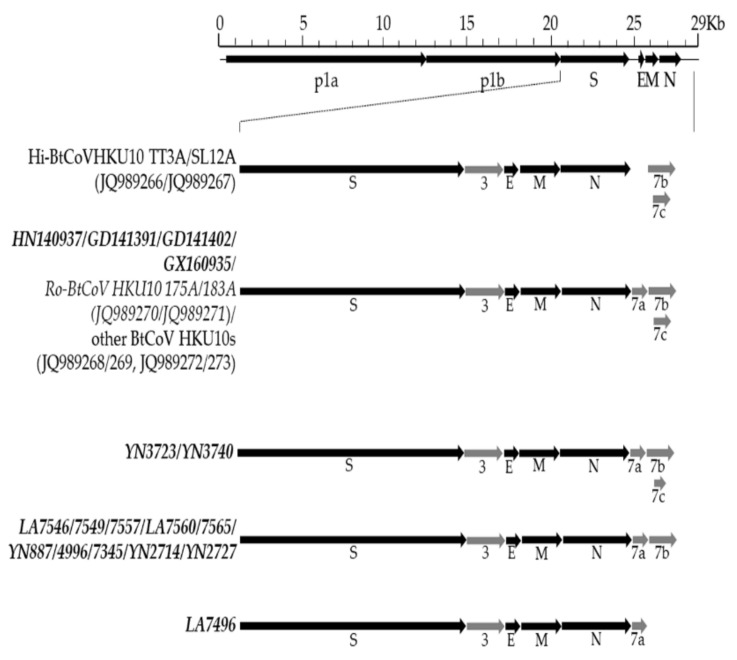
Schematic diagram of genomic organization of BtCoV HKU10 strains. The genomic regions or ORFs are described and compared in Supplementary Tables S3 and S4. Solid bars indicate conserved genes and grey letters indicate group-specific genes. Upper letters indicate structural proteins and lower letters indicate nonstructural proteins (p1a and p1b) and accessory proteins.

We then sequenced the complete genomes of 17 BtCoV HKU10 strains, considering the variations in *RdRp* sequences, bat species, and sampling location (Figure 3). These genomes ranged between 28,021 to 28,540 nt, with 38.19 to 39.81% G + C contents. All of them possessed the same putative transcription regulatory sequence (TRS) core motif as known BtCoV HKU10 except the TRS preceding ORF3 in YN2714/2727 (Table S3). The genome organization were similar to known BtCoV HKU10 (Figure 3). The major differences were located in the regions encoding accessory protein ORF7 downstream the *N* gene. In total, four types of ORF7: (1) GD141391, GD141402, GX160935, and HN140937 had similar accessory genes to known BtCoV-HKU10, which included ORF7a, ORF7b and ORF7c; (2) Strain YN3723 and YN3740 had a truncated ORF7c as a result of stop-codon substitution; (3) Strain LA7496 lost ORF7b and ORF7c; (4) the rest lost ORF7c.

The size of the 5′ untranslated regions of BtCoV HKU10 strains in *H. pomona*, *H. larvatus*, and *A. stoliczkanus* were 302, 299, and 302 nt, respectively. The replicase ORF1ab occupies 20.3 to 20.4 kb and encoded 16 putative proteins. The genomes of these viruses shared >73.3% nt sequence identities to known BtCoV HKU10. A separate deduced amino acid sequence comparison of seven conserved ORF1ab domains was performed as suggested by the International Committee on Taxonomy of Viruses (ICTV) for formal CoV species delineation. These domains possessed >90% aa identities to the prototype of *BtCoV HKU10*, suggesting that these viruses are BtCoV HKU10 variants. 

We compared the genome nt identities, structural protein and non-structural protein aa identities among these viruses (Supplementary Table S4). Based on the full-length genomic sequences, viruses from *A. stoliczkanus* and *H. pomona* shared higher similarity (>82.6% and >85.0% nt identities, respectively) to known Hi-BatCoV HKU10 than those from *H. lavartus* (<72.0% nt identities). However, viruses from the same species were more closely related based on spike protein sequences. The spike proteins of BatCoV HKU10 from *H. pomona* and *A. stoliczkanus* were more similar (>79.8% aa identities) than the ones from *H. larvatus* and *R. leschenaultia* (<70.7% aa identities). For other structural and non-structural proteins, E, M, N and NS3 were conserved, while the NS7a, NS7b and NS7c possessed low aa identities among different strains (as low as 33.3%, 21.1% and 41.3%, respectively, for NS7a, b, and c).

We further analyzed the deduced amino acid sequence of polyprotein 1ab. The putative mature nonstructural proteins (NSPs) in the ORF1ab encoding the replicase complex was predicted as previously described. Five putative cleavage sites recognized by 3C-like proteinase (3CLpro) and papainlike (PLpro) in YN2714 strain showed difference to other BtCoV-HKU10 strains. Different putative cleavage motifs were also discovered between NSP1/2 and NSP5/6 in various strains of BtCoV HKU10s (Table 1).

### 3.3. Phylogenetic Analyses of Polyprotein 1ab and Structural Proteins

**Figure 4:**
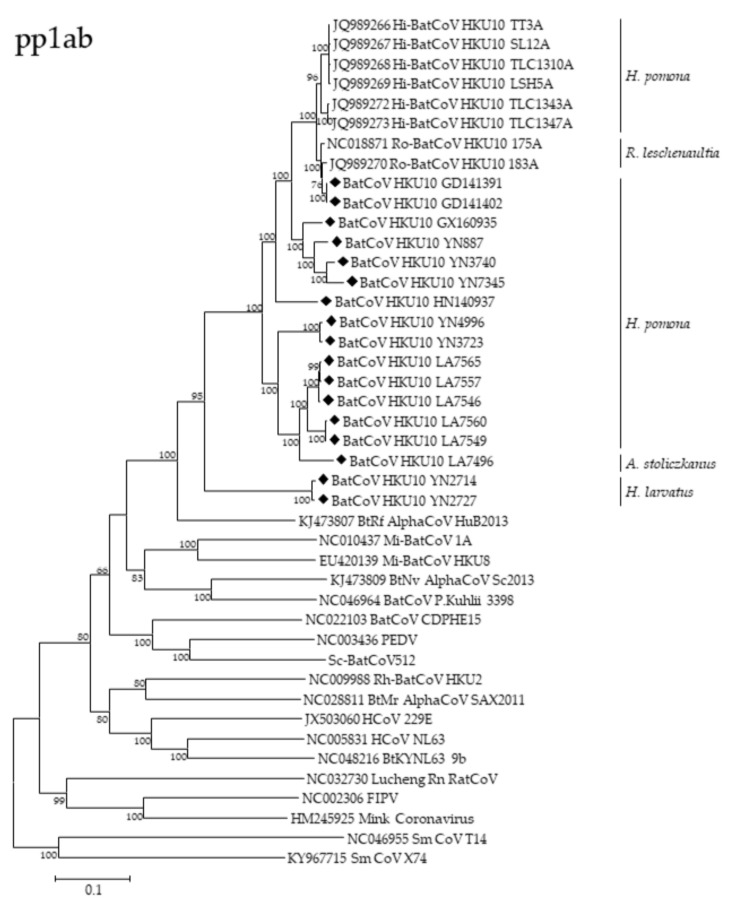

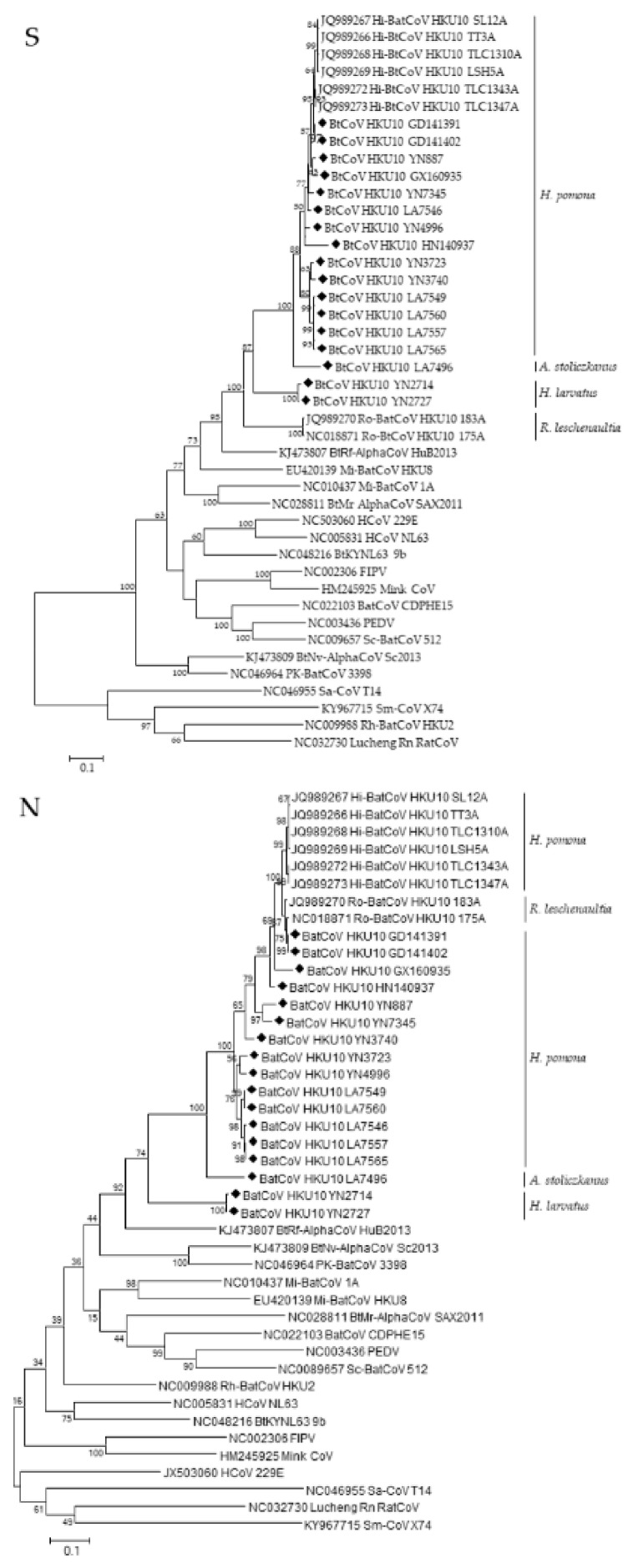

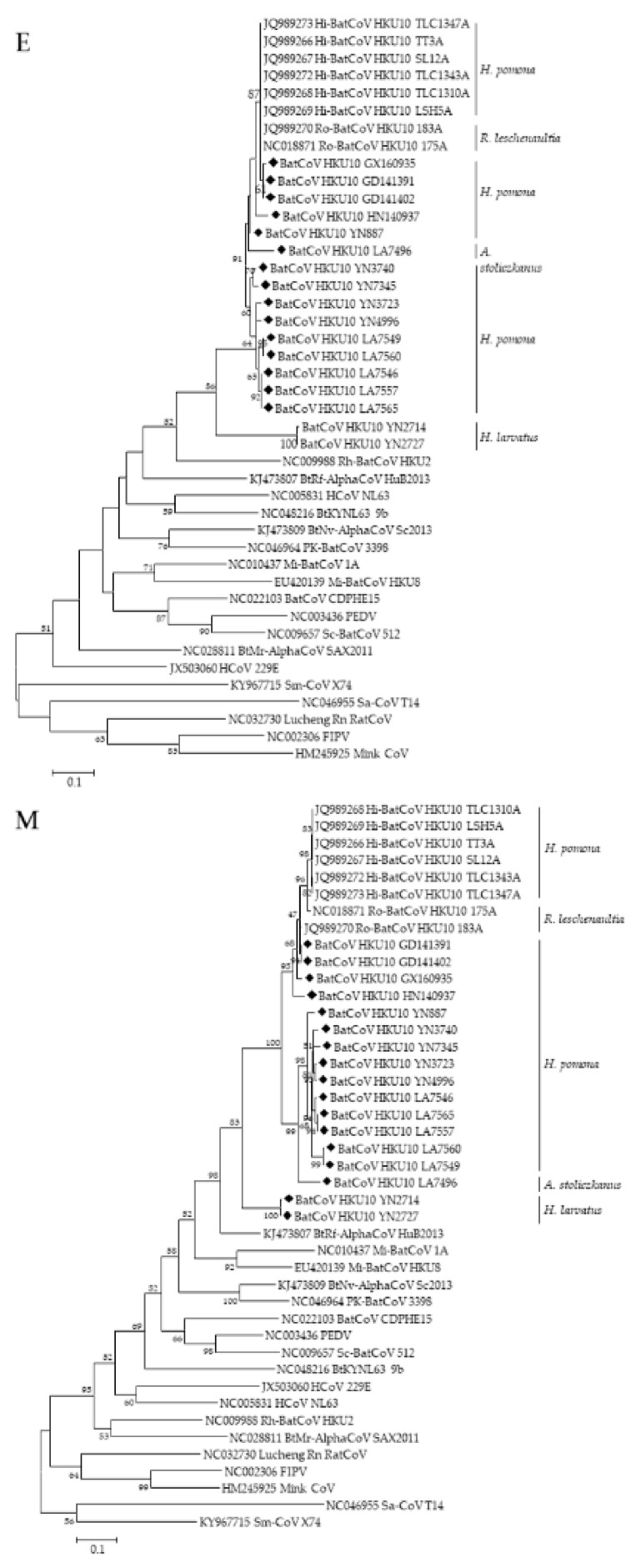
Phylogenetic trees of pp1ab (aa sequences), (S, N, M, E) (nt sequences). Trees were constructed by the maximum likelihood method using the Poisson model with bootstrap value determined with 1000 replicates. The scale bar indicates the estimated number of substitutions per 10 amino acids or nucleotides. Filled diamonds indicate viruses detected in this study.

We constructed the phylogenetic trees using the deduced aa sequences of pp1ab or nucleotide sequences of S, E, M, N genes and genomes, respectively (Figure 4, Figure S3). All the trees except the S showed similar topology and they revealed that the BtCoV HKU10s were likely phylogeographically associated. The virus strains from south China (Guangdong, Guangxi, Hainan, and Hong Kong) were always clustered together despite the fact that they were from two different bat species. However, two strains detected in Yunnan province (YN2714 and YN2727) were distantly related to other strains in all five trees, suggesting a long independent evolutionary history in *H. larvatus*. The five HKU10 strains from *H. pomona* in Yunnan province were separated into two lineages with different topology, indicating virus genetic diversity in Yunnan. LA7496 from Laos formed a relatively distinct lineage from other HKU10s detected in Laos from its different hosts. 

Remarkably, a different phylogenetic pattern was observed in the S protein tree, in which the HKU10s formed four distinct clusters which were correlated to their host species rather than sampling locations. The S proteins from *H. Pomona* were much closer to that in *A. stoliczkanus* than those in *H. larvatus*, though *H. pomona* and *H. larvatus* belonged to the same bat genus. Upon the phylogenetic tree, S protein of HKU10s in *H. pomona* could be further divided into two branches, and the virus strains in Yunnan were distributed in both branches. The viruses in each of the two branches shared high sequence similarities.

### 3.4. Recombination Analysis

**Figure 5:**
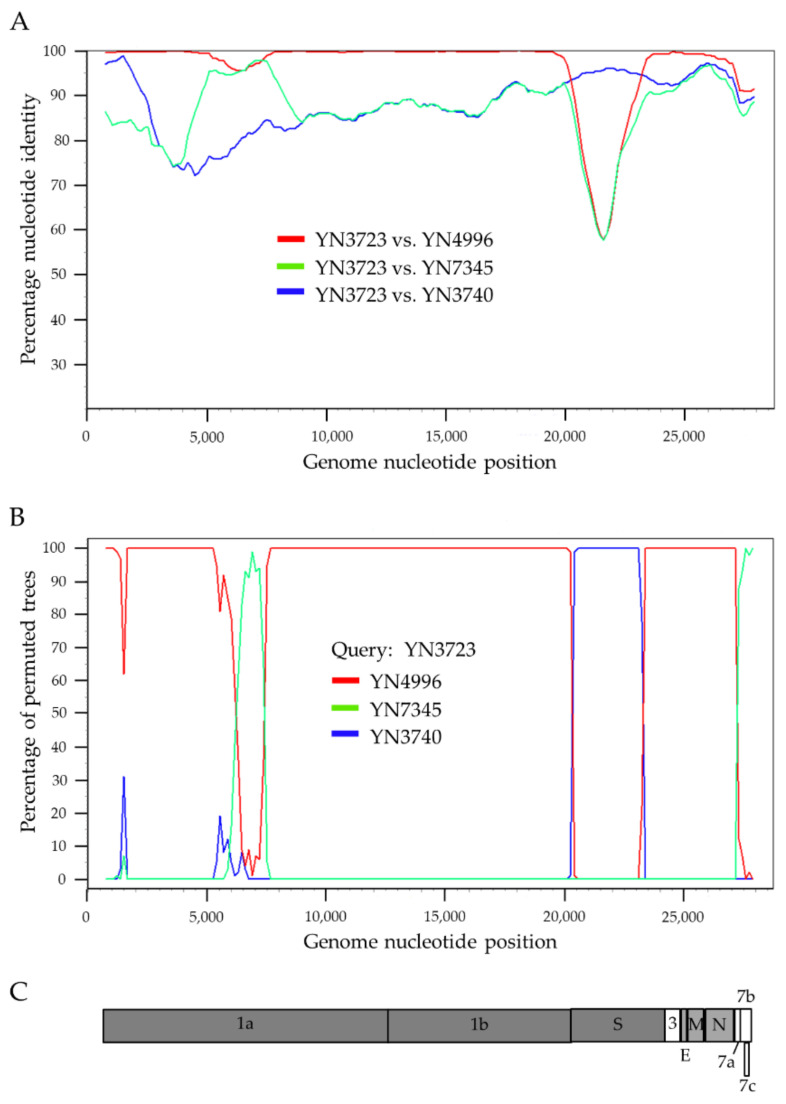
Evidence of recombination in BtCoV HKU10s. Similarity (**A**) (window of 40 nt, step size of 40 nt) and recombination (**B**) (window of 1500 nt, step size of 150 nt) plots were generated using Simplot (V3.5.1) with default settings. Full-length genome sequence of YN3723 was used as query sequence and YN3740, YN4996 and YN7345 as reference sequences. All analyses were performed with Kimura model, a window size of 1500 base pairs, and a step size of 150 base pairs. The map of query genome sequences (**C**) are used to position breakpoints.

The full-length genome sequences of BtCoV HKU10s were screened for potential recombination events as previously described. Briefly, the sequences were scanned sequentially by a bootscan algorithm and similarity plot analysis. Multiple potential recombination events were observed at ORF1a and *S* gene (Figure 5). YN3723 was likely to be a recombinant strain from three HKU10s (YN3740, YN4996, and YN7345) discovered in the same cave in Yunnan province, with strong *p*-value (<10^−32^). Breakpoints were identified in the genome of YN3723 at nt 20,275 and 23,265, the region between which encoded the C-terminal of pp1ab, S1 subunit and N-terminal of S2 subunit of S protein. In this region, the strain YN3723 was highly similar (95% nt identity) to the strain YN3740. Meanwhile, in the upstream ORF1ab sequence from nt 20,275, YN3723 displayed the highest genetic similarity (99% nt identity) to that of the strain YN4996.

Other BtCoV HKU10 were also screened for evidence of potential recombination events. However, no significant recombination breakpoint among these viruses and other HKU10 strains.

### 3.5. Estmation of Synonymous and Nonsynonymous Substitution Rates

High *Ka*/*Ks* ratios and substantial changes in the spike proteins of coronaviruses may reflect rapid viral evolution soon after introduction into a new animal host, as demonstrated in previous studies [38]. We examined the *Ka*/*Ks* ratios among BtCoV HKU10 in different hosts. A set of low ratios were observed in polymerase and structural genes of YN2714 and YN2727 (Table 2), providing another piece of evidence for the long-independent evolution history of BtCoV-HKU10 in *H. lavatus* bats. BtCoV HKU10 in *H. pomona*, *R. leschenaultii and A. stoliczkanus* possessed highly similar genome sequences (>80% sequence identity), indicating that interspecies transmission among these three bat species occurred recently, with subsequent viral adaption in the new host species [25]. To test the hypothesis, we then determined the *Ka/Ks* ratio for the various coding regions in HKU10s of different host species (Table 2). Higher *Ka/Ks* ratios were observed in Ro-BtCoV HKU10 *S* (0.382) than in other HKU10s (0.229–0.273). Higher *Ka*/*Ks* ratios were also observed in ORF7a, 7b, and 7c genes. The *Ka*/*Ks* ratio of *7b* genes from *H. pomona*, *H. larvatus* and all HKU10s was 1.037, 1.161, and 0.951, respectively, which suggested that the *7b* gene was likely under positive selection in these two clades.

### 3.6. Estimation of Divergence Date

**Figure 6:**
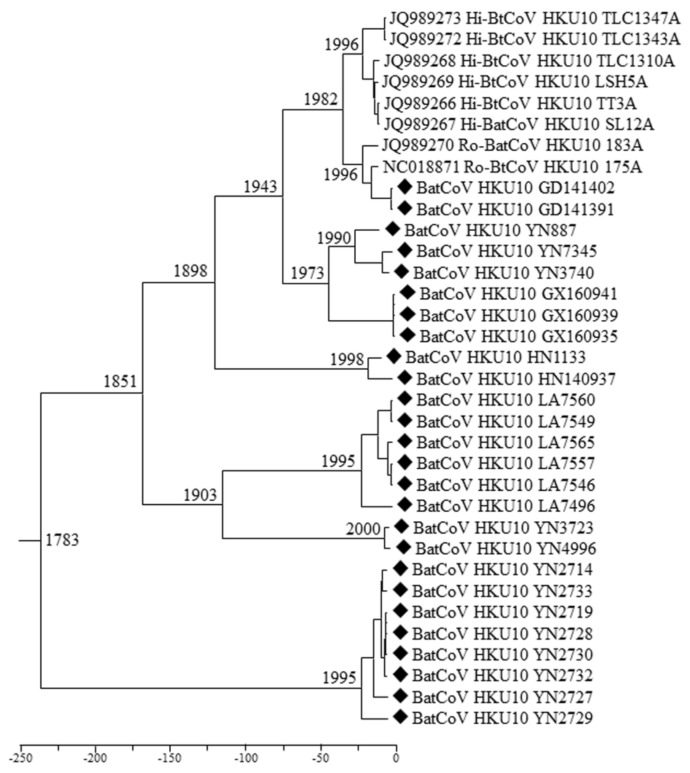
Estimation of the tMRCA of BtCoV HKU10. The time-scaled phylogeny was summarized from all MCMC phylogenies of the RdRp gene data set analyzed under the relaxed-clock model with an exponential distribution (Uced) in BEAST version 2.4.4. Filled diamonds indicate viruses detected in this study.

We used relaxed clock model with UCed on *RdRp* sequences to calculate the divergence dates of BtCoV HKU10. The most recent common ancestor (MRCA) of BtCoV HKU10 was estimated at 1783 (HPDs, 1410.72-1981.43), approximately 238 years ago. For HKU10s in different host species, the MRCA of in *H. pomona*, *R. leschenaultia*, *H. larvatus* and *A. stoliczkanus* were estimated at 1851 (HPDs, 1681.82-1982.84), 1996 (HPDs, 1979.87-2004.09), 1995 (HPDs, 1965.80-2009.92), and 1995 (HPDs, 1958.63-2012.47), respectively (Figure 6). The estimated mean substitution rate of the RdRp data set was 3.705 × 10^−4^ substitution per site per year, which is comparable to previous estimations for other CoVs [25,39,40].

### 3.7. BtCoV HKU10 Spike-Mediated Pseudovirus Entry

**Figure 7:**
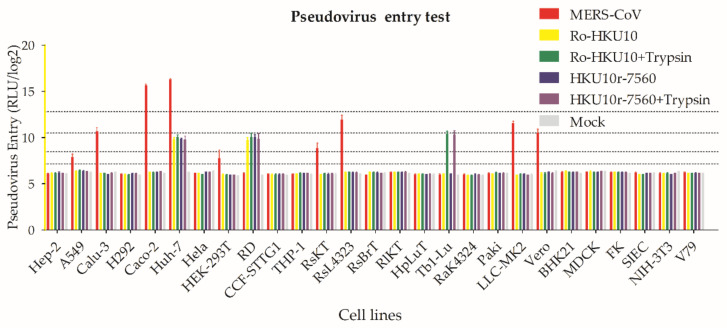
HKU10 spike-mediated pseudovirus entry. Ro-BtCoV HKU10, LA7560, and MERS-CoV spike-mediated pseudovirus were produced to screen their cell tropism. For Trypsin treatment, pseudoviruses were incubated with 50 μg/mL TPCK-treated trypsin at room temperature for 10 min, and then add 50 μg/mL soybean trypsin inhibitor to stop the reactions. Trypsin treated or untreated pseudoviruses were added to cells and incubated. Then, 48 h post infection, cells were lysed and luciferase were detected.

To understand the infectivity of BtCoV HU10, we constructed pseudotyped lentiviruses carrying spike proteins from Ro-BtCoV HKU10 (NC018871) and LA7560, respectively. A total of 27 cell lines from human, bat, and other mammals were challenged. Huh-7 (human lung) and RD (human muscle) cells are susceptible to these viruses with and without treatment of trypsin. Pseudovirus could enter Tb1-Lu (bat lung) cell only when cells were treated with trypsin. HKU10 seemed unable to infect respiratory cells. As previously reported, the pseudovirus assay for MERS-CoV spike could enter A549, Calu-3, Caco-2, Huh-7, HEK-293T, LLC-MK2, and Vero cells [41,42] (Figure 7 and Table S5).

## 4. Discussion

In this study, we screened for BtCoV HKU10 from our archived 8004 bat fecal samples collected in 25 provinces of China. We found twenty-six (4.7%) BtCoV HKU10 strains in three bat species (547 bats) from more habitats including Guangdong, Guangxi, Hainan and Yunnan Province of China, and the Louang Namtha Province of Laos. Combining previous reports in Guangdong Province and Hong Kong in China, Thailand and Laos, we further demonstrate that BtCoV HKU10 are widely spread in south and southwest China and southeast Asia. Two bat species, *A. stoliczkanus* and *H. larvatus*, were firstly identified as the host of BtCoV HKU10. Our results expand the host ranges and geographic distributions of BtCoV HKU10.

The genomes of BtCoV HKU10, both previously and newly discovered are highly similar except in the *S* genes and accessory genes *ORF7*. Analyses based on the most conserved regions (*ORF1ab* and *RdRp* genes) indicate that the newly discovered viral strains together with previously described strains can be classified into eight lineages which are closely associated with their sampling locations. The previously reported BtCoV HKU10 strains from Guangdong and Hong Kong are closely related, although the two strains were found in two members of different bat suborders: *H. pomona* and *R. leschenaultia*. In our study, viruses from Yunnan belong to three lineages and the one found in *H. larvatus* is distantly related to BtCoV HKU10 found in *H. pomona*, suggesting an another interspecies transmission event of BtCoV HKU10. Previous studies suggested that the BtCoV HKU10 transmitted from *R. leschenaulitia* to *H. pomona*. However, in our extensive surveillances, we only found BtCoV HKU10 among *Hipposideros* bats, especially in *H. pomona*. We suggest that *H. pomona*, not *Rousettus* bats, are the primary reservoirs of BtCoV HKU10 based on the following reasons: first, the richest divergence, wider geographic distribution and longer independent evolution history of BatCoV HKU10 were observed in *Hipposideros* hosts; second, the *Ka*/*Ks* ratio of the previously reported S gene in *R. leschenaultia* (0.382) is higher than that from other hosts (0.229–0.273), which indicate a higher overall selection pressure and changing in Ro-BatCoV HKU10 spike; third, with previously described method, the molecular-clock analysis of all available *RdRp* genes dated the MRCA of all BatCoV HKU10 strains at 1783 (HPDs, 1410.72–1981.43). The MRCA of *H. pomona* and *R. leschenaultia* were estimated at around 1851 (HPDs, 1681.82–1982.84) and 1996 (HPDs, 1979.87–2004.09), respectively, indicating a recent interspecies transmission from *H. Pomona* to *R. leschenaultia*.

The major differences of BtCoV HKU10 from different hosts and locations were found in their S proteins. As we failed to cultivate BtCoV HKU10 in vitro, we used spike-pseudotyped lentivirus system for cell-entry and host adaptation studies. We screened 27 cells form human, bat and other mammals and found that the tested BtCoV HKU10 could infect multiples cell lines including human and bat species, providing evidence of potential interspecies transmission at cell-entry stage. Coronaviruses demonstrate a complex pattern for receptor recognition as both protein and sugar at cell surface could be used as cellular receptor mediating virus entry. The sugar-receptor-binding region is located at the N-terminal domain (NTD) of S1 region [43,44,45], while the protein-receptor-binding domain is mainly located at the C-terminal domain (CTD) of S1 domain [43,46,47,48]. Protease cleavage triggers for membrane fusion by coronavirus spike protein, which is another determining factors for coronavirus entry. The host proteases, including proprotein convertases, extracellular proteases, cell surface proteases, and lysosomal proteases cleave coronaviruses at different stages during virus maturation and entry [49,50]. In this study, we detected the spike protein of the pseudoviruses produced by 293T cells with Western blotting and found no evidence for human proprotein convertases cleavage (Figure S2). The mature pseudo-typed virions with intact spike provide an evidence that human proprotein convertases may not participate in the BtCoV HKU10 spike maturation just as SARS-CoV [51]. 

Bats harbor a vast number of coronaviruses. The ability to fly, their worldwide distribution and intimate contact with human and other domestic animals provide bats with opportunities for virus spillover from their natural reservoirs to human society, as demonstrated by the SARS and MERS outbreaks. With increasing genetic information about bat coronaviruses, bats are considered as the natural reservoirs of most alpha- and beta-coronaviruses. However, little information was known regarding the risk of their interspecies transmission. Further investigations will be focused on virus isolation, receptor usage and the pathogenesis of these CoVs, which will provide further information for predicting the potential spillover of these viruses. 

## Figures and Tables

**Table 1:** Characteristics of predicted nonstructural proteins of ORF1ab in different strains of BtCoV HKU10 in this study.

NSP	Putative Functional Domain(s)	YN2714			LA7496			YN887			GD141391		
		Amino Acids Position in ORF1ab	Predicted Size (aa of Protein)	C-End Predicted Cleavage Site	Amino Acids Position in ORF1ab	Predicted Size (aa of Protein)	C-End Predicted Cleavage Site	Amino Acids Position in ORF1ab	Predicted Size (aa of Protein)	C-End Predicted Cleavage Site	Amino Acids Position in ORF1ab	Predicted Size (aa of Protein)	C-End Predicted Cleavage Site
NSP1	Unknown	M1-A195	195	NA|GP	M1-A195	195	VA|KP	M1-A195	195	VA|KP	M1-A195	195	VA|KV
NSP2	Unknown	G196-G888	693	TG|GG	K196-G888	693	RG|SG	K196-G888	693	RG|SG	K196-G888	693	RG|SG
NSP3	ADRP, PL2 pro	G889-G2463	1575	CG|SG	S889-G2529	1641	CG|SG	S889-G2523	1635	CG|SG	S889-G2518	1630	CG|SG
NSP4	Hydrophobid domain	S2464-Q2941	478	LQ|AG	S2530-Q3007	478	LQ|SG	S2524-Q3001	478	LQ|SG	S2519-Q2996	478	LQ|SG
NSP5	3CL pro	A2942-Q3243	302	LQ|ST	S3008-Q3309	302	LQ|ST	S3002-Q3303	302	LQ|SN	S2997-Q3298	302	LQ|SN
NSP6	Hydrophobid domain	S3244-Q3519	276	VQ|SK	S3310-Q3585	276	VQ|SK	S3304-Q3579	276	VQ|SK	S3299-Q3574	276	VQ|SK
NSP7	Replicase	S3520-Q3602	83	LQ|SV	S3586-Q3668	83	LQ|SV	S3580-Q3662	83	LQ|SV	S3575-Q3657	83	LQ|SV
NSP8	Replicase	S3603-Q3797	195	LQ|NN	S3669-Q3863	195	LQ|NN	S3663-Q3857	195	LQ|NN	S3658-Q3852	195	LQ|NN
NSP9	Replicase	N3798-Q3905	108	LQ|AG	N3864-Q3971	108	LQ|AG	N3858-Q3965	108	LQ|AG	N3853-Q3960	108	LQ|AG
NSP10	RNA synthesis protein	A3906-Q4041	136	VQ|AF	A3972-Q4108	137	MQ|AF	A3966-Q4102	137	MQ|AF	A3961-Q4097	137	MQ|AF
NSP11	Unknown (short peptide at the end of ORF1a)	A4042-N4058	17		A4109-N4125	17		A4103-N4119	17		A4098-N4114	17	
NSP12	RdRp	A4042-Q4968	927	LQ|AA	A4109-Q5035	927	LQ|SA	A4103-Q5029	927	LQ|SA	A4098-Q5024	927	LQ|SA
NSP13	Hel, NTPase	A4969-Q5565	597	LQ|AG	S5036-Q5632	597	LQ|AG	S5030-Q5626	597	LQ|AG	S5025-Q5621	597	LQ|AG
NSP14	ExoN, NMT	A5566-Q6083	518	LQ|SL	A5633-Q6150	518	LQ|SL	A5627-Q6144	518	LQ|SL	A5622-Q6139	518	LQ|SL
NSP15	NeudoU	S6084-Q6422	339	LQ|SA	S6151-Q6487	337	LQ|SA	S6145-Q6483	339	LQ|SA	S6140-Q6476	337	LQ|SA
NSP16	2′-O-MT	S6423-K6724	302		S6488-C6799	312		S6484-K6785	302		S6477-H6783	307	

**Table 2:** Estimation of nonsynonymous and synonymous substitution rates in the genomes of BtCoV HKU10 in different host species.

Genes	*Ka*/*Ks* Ratio				
	BtCoV HKU10	BtCoV HKU10	BtCoV HKU10	BtCoV HKU10	BtCoV HKU10
	(*H. pomona*, 20 Strains)	(*A. stoliczkanus,* 1 Strain)	(*H. lavatus*, 2 Strains)	(*R. leschenaultia*, 2 Strains)	(All, 25 Strains)
NSP1	0.354	0.214	0.237	0.150	0.297
NSP2	0.309	0.131	0.391	0.171	0.403
NSP3	0.359	0.190	0.303	0.139	0.370
NSP4	0.189	0.104	0.225	0.059	0.274
NSP5	0.176	0.033	0.205	0.003	0.221
NSP6	0.317	0.167	0.097	0.053	0.477
NSP7	0.265	*Ka* = 0, *Ks* = 0.035	*Ka* = 0, *Ks* = 0	*Ka* = 0, *Ks* = 0	0.166
NSP8	0.174	0.500	0.010	0.012	0.287
NSP9	0.198	*Ka* = 0, *Ks* = 0	0.073	0.031	0.598
NSP10	0.200	*Ka* = 0, *Ks* = 0.032	0.120	0.056	0.138
NSP11	*Ka* = 0, *Ks* = 0.066	*Ka* = 0, *Ks* = 0.131	*Ka* = 0, *Ks* = 0.066	*Ka* = 0, *Ks* = 0	1.176
NSP12	0.123	0.053	0.092	0.012	0.067
NSP13	0.112	0.161	0.087	0.008	0.0738
NSP14	0.081	0.052	0.142	0.034	0.109
NSP15	0.127	0.114	0.086	0.026	0.167
NSP16	0.103	0.117	0.126	0.114	0.105
S	0.240	0.243	0.272	0.382	0.259
ORF3a	0.069	0.169	*Ka* = 0, *Ks* = 0	0.051	0.274
E	0	0.204	0.227	*Ka* = 0, *Ks* = 0	0.218
M	0.192	0.024	0.147	0.275	0.066
N	0.151	0.188	0.218	0.146	0.207
ORF7a	0.339	0.403	0.442	0.336	0.662
ORF7b	1.0368	NA	1.161	0.136	0.951
ORF7c	0.630	NA	NA	0.609	0.4433

## Data Availability

The data presented in this study are openly available under accession numbers MN477899 to MN477915.

## Supplementary Materials

The following are available online at https://www.mdpi.com/article/10.3390/v13101962/s1, Figure S1: Strategy of BatCoV HKU10 genomes sequencing. Figure S2: Characterization of BtCoV HKU10 spike mediated pseudovirus. Figure S3: Phylogenetic tree of BatCoV HKU10. Table S1: Primer pairs for HKU10 detection and genome sequencing. Table S2: Detection of HKU10 in bats by RT-PCR. Table S3: Genome size, coding potential, and putative transcription regulatory sequences of 11 BtCoV HKU10 strains. Table S4: Comparison of whole genomes (nt) and coding regions (aa) of representation strains of BtCoV HKU10. Table S5: Susceptibility test of Ro-BtCoV HKU10 and BtCoV HKU10 LA7560.
