# Supplementary material for: Genomic Characterization of Diverse Bat Coronavirus HKU10 in Hipposideros Bats
Source: Viruses. 2021 Sep 29;13(10):1962. doi: 10.3390/v13101962 (PMC8540636; doi:10.3390/v13101962)
Supplement: Supplementary file 1 [file viruses-13-01962-s001.zip › viruses-1340913-supp-proof/viruses-1340913-supp-conv.pdf]

viruses MDPI

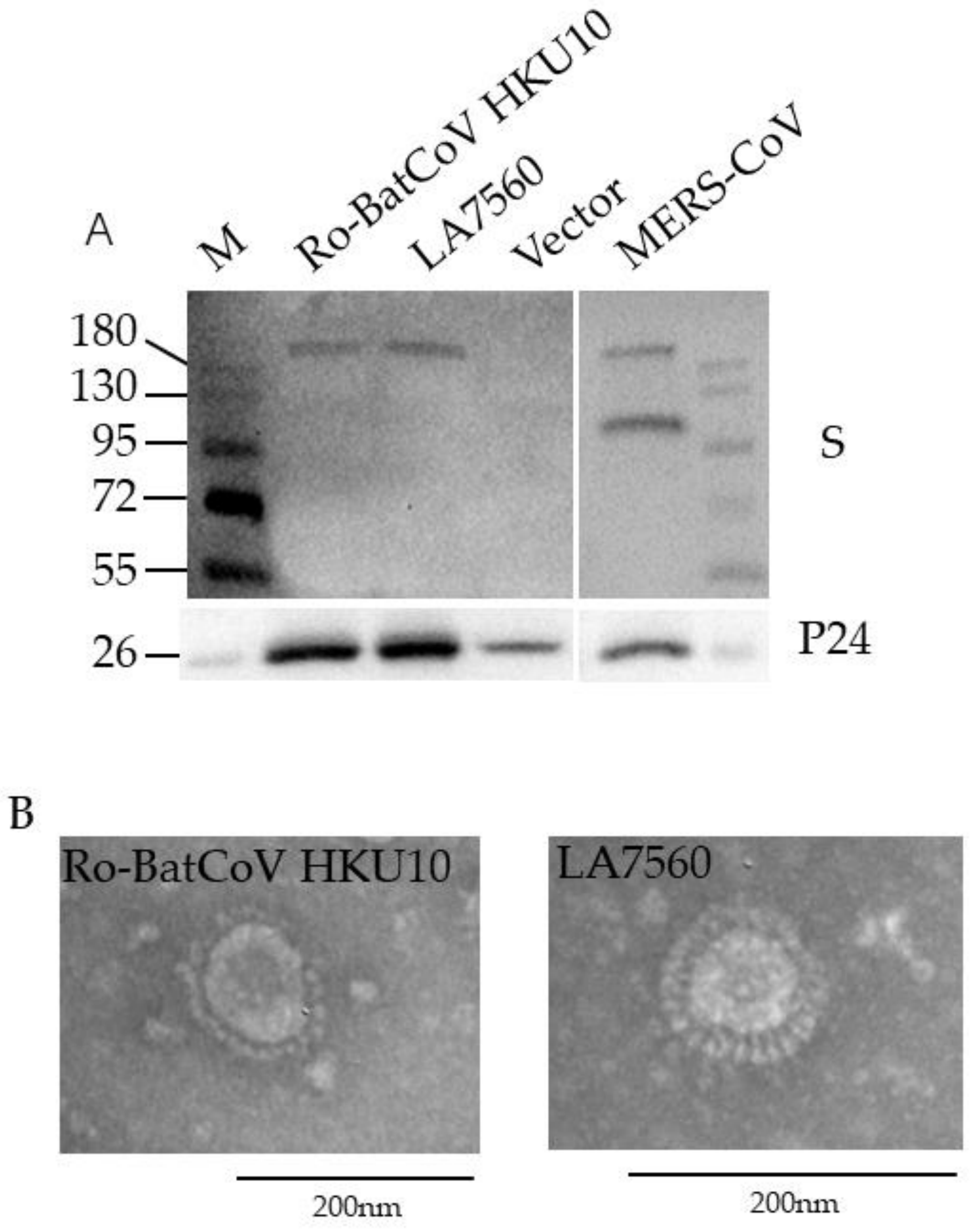

Figure S1. Characterization of BatCoV HKU10 spike mediated pseudovirus. (**A**) Western blot analysis of the protein expression of pseudoviruses in the medium supernatant. (**B**) Electron-microscopical observation of negative-stain coronavirus particles. The bars represent 200nm.

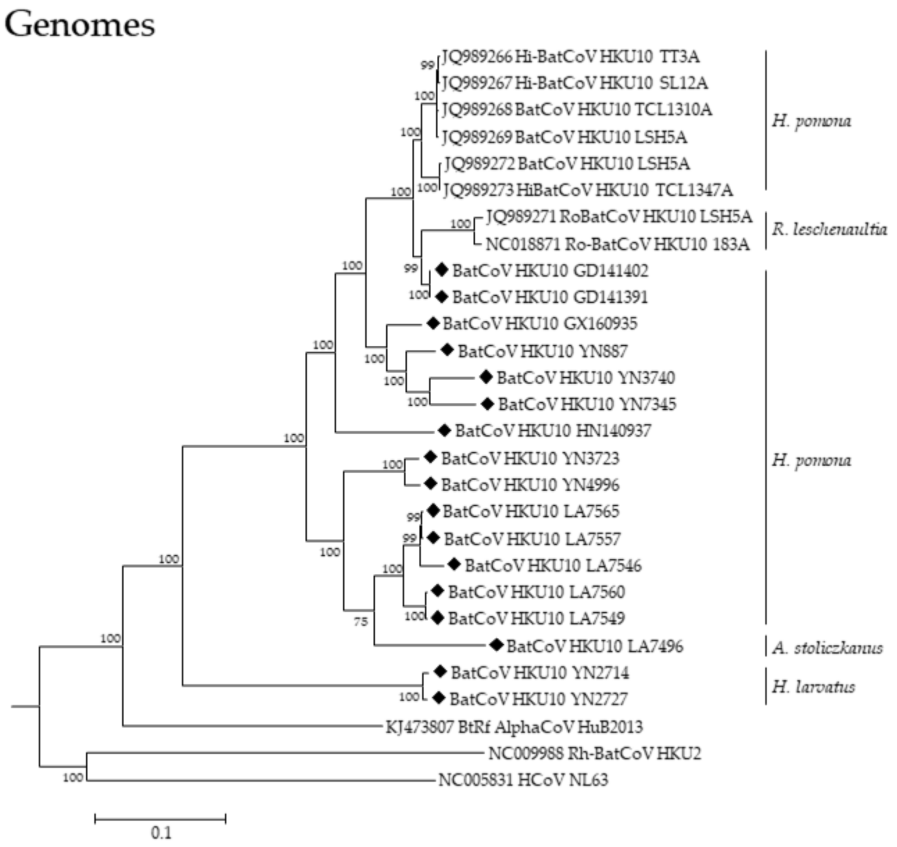

Figure S2. Phylogenetic tree of BatCoV HKU10. The tree was constructed using genomes with 5′ and 3′ UTR removed based on maximum likelihood method, with bootstrap value of 1000 replicates. The scale bar indicates the estimated number of substitutions per 10 nucleotides. Filled diamonds indicate viruses detected in this study.

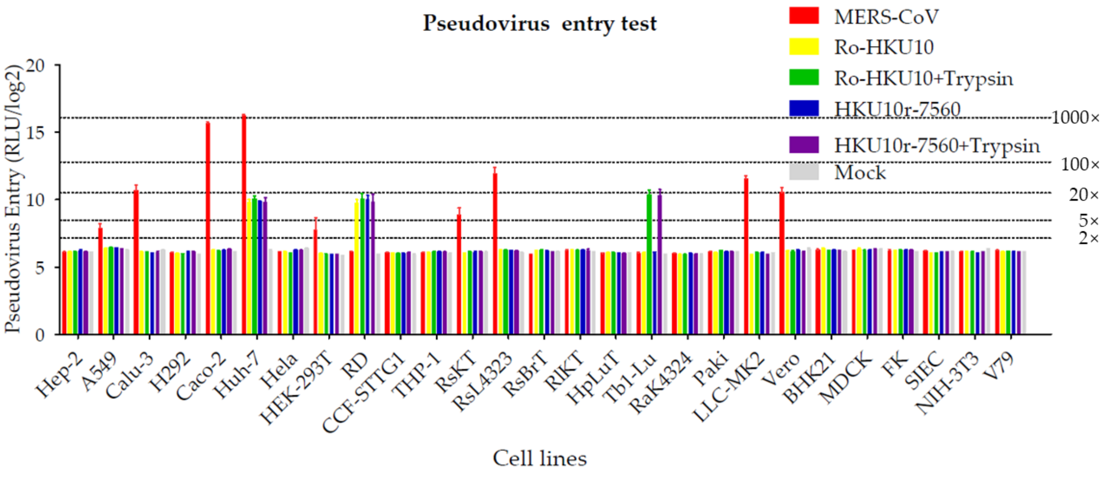

Figure S3. HKU10 spike-mediated pseudovirus entry. Ro-BtCoV HKU10, LA7560, and MERS-CoV spike-mediated pseudovirus were produced to screen their cell tropism. For Trypsin treatment, pseudoviruses were incubated with 50 μg/mL TPCK-treated trypsin at room temperature for 10 min, and then add 50 μg/mL soybean trypsin inhibitor to stop the reactions. Trypsin treated or untreated pseudoviruses were added to cells and incubated. 48h post infection, cells were lysed and luciferase were detected.
